# Blood protein biomarkers in hepatocellular carcinoma: progress and challenges

**DOI:** 10.7717/peerj.21196

**Published:** 2026-04-29

**Authors:** Lu Zhang, Mingjing Zhang, Yingying Bao, Jiajia Yang, Tingting Luo, Yan Zhang, Yange Wang, Peijie Liu, Xiangqian Guo

**Affiliations:** 1 Department of Public Health and Epidemiology, School of Basic Medical Sciences, Henan University, Kaifeng, China; 2 Henan Provincial Engineering Center for Tumor Molecular Medicine, Kaifeng, China; 3 Department of Oncology, Kaifeng Central Hospital, Kaifeng, China; 4 Department of Cell Biology and Genetics, School of Basic Medical Sciences, Henan University, Kaifeng, China

**Keywords:** Autoantibodies, Blood protein, Hepatocellular carcinoma, Tumor antigens

## Abstract

Hepatocellular carcinoma (HCC) is an important cause of cancer-related death. Due to the lack of typical clinical symptoms in early-stage HCC, most HCC patients are diagnosed at an advanced stage and have lost the opportunity of surgery, which results in a poor prognosis. Therefore, early detection and timely intervention are the most effective methods to reduce the mortality of HCC. Blood flows through different organs and tissues, and there are abundant tumor biomarkers in blood, which can provide real-time information for the early diagnosis and prognostic prediction of HCC, so blood tumor biomarkers have become an effective supplement to imaging technology. As the most ideal biomarkers for disease diagnosis, serum and plasma proteins have been the main focus for biomarker development. In this review, we summarized the research progress of potential blood protein biomarkers (tumor-associated antigens and tumor-associated autoantibodies) in HCC and discussed their obstacles in clinical translation.

## Introduction

Liver cancer is the sixth most common cancer worldwide and the third leading cause of cancer-related death (Bray et al., 2024). Hepatocellular carcinoma (HCC) is the most predominant pathological type, accounting for approximately 90% of liver cancer cases (Llovet et al., 2021). The biological processes involved in the occurrence and development of HCC are complex. When the balance between tumor suppressor genes and oncogenes is disrupted, it will lead to abnormal activation of downstream signaling pathways, and further result in abnormal differentiation and proliferation of hepatocytes and neovascularization (Wang & Deng, 2023).

Currently, surgery remains the primary treatment method for achieving long-term survival in HCC. Due to the lack of typical clinical symptoms for early-stage patients, HCC usually progressed into the advanced stage by the time of diagnosis, and only 30–40% of patients were suitable for surgical treatment (Wang et al., 2023), while systemic anti-tumor therapy is needed for patients with advanced HCC. Therefore, more effective biomarkers for early diagnosis and targeted therapy are identified as priorities to improve the 5-year survival rate of HCC patients.

Proteins are functional molecules in the regulation of biological processes and are the most ideal biomarkers for disease diagnosis. Blood contains a rich proteome originating from the organs of all over the body by circulation system, it could reflect the physiological or pathological state of original tissues. The circulation nature of blood allows for the dynamically monitoring disease progression in real time, while the minimally invasive nature of blood sampling makes it more acceptable to patients, which is crucial for its widespread application in clinical evaluations (Huang et al., 2022). Circulating biomarkers have important roles in clinical diagnosis, disease monitoring, and prognosis evaluation.

Tumor markers are a class of substances produced by tumor cells during the initiation and progression of tumors, or by the host’s response to the stimulation of tumors (Filella, Rodríguez-Garcia & Fernández-Galán, 2022). Tumor markers include carcinoembryonic antigen, proto-oncogenes and oncogenes, enzymes and isozymes, hormones, receptors, glycoprotein antigen, cytokines, *etc* (Zhou et al., 2024; Zhao, Ju & Li, 2013). Although identifying “ultra-early biomarkers” is the ultimate goal for preventing HCC, finding “pre-neoplastic state” biomarkers is highly challenging. Firstly, the number of abnormal cells is very small in the precancerous lesions (*e.g.*, nodules of cirrhosis, atypical hyperplasia) or in the extremely early stage of cancer cells, and concentration of biomarkers released by them into the blood is extremely low, which is very difficult to detect in the huge “background noise”. Secondly, the etiology of HCC is complex, the precancerous state is long and unstable, and the biomarkers need to have a very high “predictive specificity”. Finally, not all abnormal cells will release internal change signals into the peripheral blood. At present, it is not yet fully clear in which stage biomarkers are released and through what mechanism (Sun et al., 2024; Xing et al., 2023a).

With the development of high-throughput proteomics techniques, hundreds of proteins have been identified in the blood of HCC patients as biomarker candidates in recent decades. The most popular blood protein biomarkers for HCC are tumor-associated antigens (TAAs) and tumor-associated antibodies (TAAbs) in the past years. In this review, we explore the predictive performance and application scope of these blood protein biomarkers, and summarize the latest research results and future development prospects.

## Survey Methodology

From January 1972 to December 2024, we conducted the literature search using the following strategies: (1) (blood OR circulation) AND biomarkers AND hepatocellular carcinoma; and (2) (proteins OR proteomics) AND biomarkers AND hepatocellular carcinoma in the Pubmed and Web of Science. A total of 31,721 publications were retrieved, and 21,878 publications remained after deduplication. Research and review articles in the English language were only included in this review. The titles and abstracts of the literature were first screened preliminarily, and then the full-text articles were obtained for further evaluation. The exclusion criteria for the literature are: (1) Studies not using clinical samples. (2) Literature that does not provide exact diagnostic and prognostic analysis results such as sensitivity, specificity, risk ratio (HR), *etc*. (3) Commonly used HCC biomarkers such as AFP, DCP and so on have been reported alone or in combination with other biomarkers in a large number of published literatures, and these biomarkers are not described in detail again in this study. (4) Research on clinical common detection indicators such as CRP, beta 2-microglobulin, D-Dimer, *etc*. These proteins are usually produced and secreted by liver or are affected by liver function. (5) For literature describing the same biomarker, exclude those with insufficiently detailed research results or small sample sizes (<30 cases). In addition, the references of relevant literature were searched to include more eligible studies. Therefore, this study mainly focused on new potential blood biomarkers for HCC. Finally, 95 articles were included in this study.

## Tumor Antigens

Tumor antigens are proteins or glycosylated proteins, glycolipids, or carbohydrates expressed on the surface of tumor cells or secreted from tumor cells or metabolites of tumor cells. After tumor antigens are released into the bloodstream, the diagnosis and following progress of tumor can be monitored in non-invasive and economic way. In addition, the response to treatment of tumor patients could be predicted as well. Tumor antigens include both tumor-specific antigens (TSAs) restricted to tumor cells and tumor-associated antigens (TAAs) present on both tumor cells and normal cells (Andersen, 2023).

### Tumor-specific antigens

TSAs, also termed neoantigens, are generated in somatic cells due to genomic mutations, abnormal transcription mutations, post-translational modification (PTM) mutations, or virus-encoded open reading frame (ORF) mutations (Li et al., 2023). The accumulation of somatic mutations in the tumor genome can serve as a key indicator of response to immune checkpoint inhibitors (ICI) therapies and these mutations are quantified and defined as tumor mutational burden (TMB). Therefore, TSAs are most often present in tumor types with a high mutation rate, such as melanoma, lung cancer and bladder cancer (Camarena et al., 2024). As we know, HCC is a tumor with a low mutation frequency and thus the mutated neoepitopes are rarely detected. However, repeated hepatic inflammation caused by host immune response during chronic hepatitis B virus (HBV) or hepatitis C virus (HCV) infection can lead to hepatic fibrosis and accelerate the turnover rate of hepatocytes, promoting the accumulation of mutations, thereby causing HCC.

Unlike normal cell antigens, neoantigens are alloantigens to the immune system, thus becoming highly specific tools for immunotherapy (Aggeletopoulou, Pantzios & Triantos, 2025). Neoantigen-based vaccines could be used as promising personalized approaches for HCC treatment. The oncogenic viral antigens of HBV-associated HCC are foreign to the body and expressed only by cancer cells. They also belong to TSAs and are very suitable as a cancer prevention vaccine (Rus Bakarurraini et al., 2020). So vaccines based on TSAs can be divided into preventive vaccines and therapeutic vaccines. Preventive vaccines reduce the incidence of cancer by selectively targeting the carcinogenic pathogens, while therapeutic vaccines are designed to attack and eliminate cancer cells by inducing immune system activation.

However, there is a most core and thorny challenge in the development of neoantigen vaccines is the selection pressure induced by treatment and immune escape. Therapeutic vaccines can eliminate tumor cells with specific neoantigens, but they also inadvertently select for variant cell clones that survive, ultimately leading to disease recurrence. Therefore, increasing antigen breadth, targeting essential antigens, combining treatments, dynamically monitoring and intervening to exert a “dynamic, sustained, multi-specific” immune pressure on tumors may be able to block tumor immune escape (Tojjari et al., 2023; Yarchoan et al., 2024).

### Tumor-associated antigens

TAAs are short polypeptides that originate from proteasome-mediated degradation of proteins from cancer cells during autophagy or following their engulfment by phagocytic cells. They can also be overexpressed or re-expressed normal self-proteins (Lu et al., 2021). Most importantly, TAAs can be detected before the clinical diagnosis of cancer. Therefore, for tumors with a lower TMB, such as HCC, immunotherapy against TAAs is necessary.

### Comman TAAs

Several serum protein biomarkers have been proposed for the diagnosis of HCC, either alone or in combination, which include AFP, AFP bound to Lens culinaris agglutinin (AFP-L3), des-gamma carboxyprothrombin (DCP) and Golgi protein 73 (GP73). However, the sensitivity and specificity of these biomarkers limit their utility for HCC surveillance (Yu & Ma, 2024). AFP is the first recognized oncofetal biomarker in HCC and plays an important role in the diagnosis, prognosis, and treatment response of HCC. The newly identified serum biomarkers candidates for HCC are mostly compared with AFP to see if they are superior to AFP, and then will be used alone or in combination with AFP (Yeo et al., 2024). Since the AFP levels of non-viral liver cancer patients are often low, thus a large proportion of early-stage non-viral liver cancer patients do not show elevated levels of AFP, and they could not be identified by measuring AFP. Therefore, novel biomarkers are needed for the screening and diagnosis of non-viral liver cancer patients.

### Other tumor-associated proteins

In recent years, many new blood protein biomarkers have been reported, and they are often overexpressed in tumor tissues or tumor cells. These biomarkers showed diagnostic and prognostic potential alone or in combination as a panel in HCC (Table 1). Most of these biomarkers were identified from serum by enzyme-linked immunosorbent assay (ELISA). ELISA is often preferred due to its simple operation, strong specificity and high sensitivity, which can accurately and reliably quantify peptide and protein molecules. These biomarkers can be used to assist in the diagnosis of HCC, even early HCC (14-3-3 beta, ANXA3, Annexin A2, AXL, CKAP4, EGFL7, Thioredoxin, *etc*.) or small size HCC (Vimentin and TGFβ1) , HBV-related HCC (VWF, CCL15, PTX3, ITIH4, *etc*.), HCV-related HCC (CCL20, DKK1, MDK, PNA, et al.) and AFP-negative HCC (AXL and LTBP2).

**Table 1:** Tumor-associated proteins identified for diagnosis of HCC.

Biomarkers	Source	Sample origin	*N*	Sen (%)	Spe (%)	AUC	Cutoff	Method
AFU (Xing et al., 2019a)	Serum	China	512	56.15	69.2	0.68	24 U/L	ARCHITECT immunoassay
AKR1B10 (Ye et al., 2019)	Serum	China	519	72.7	95.7	0.896	267.9 pg/mL	Time-resolved fluorescent assay
Annexin A2 (Sun et al., 2013)	Serum	China	218	83.2	67.5	0.79	17.3 ng/μ L	ELISA
AXL (Song et al., 2020)	Serum	China	320	95	73.3	0.888	1,202 pg/mL	ELISA
CA125 (Lopez, Balasegaram & Thambyrajah, 1996)	Serum	Malaysia	133	92	48.5	–	Male 12 U/mL Female 55 U/mL	Non-isotopic immunoassay
CCL15 (Li et al., 2013b)	Serum	China	75	88.2	93	0.964	16 ng/mL	ELISA
CCL20 (Soliman et al., 2012)	Serum	Egypt	75	83.3	93.3	–	54 pg/mL	ELISA
CKAP4 (Wang et al., 2018c)	Serum	China	400	79	67	0.821	250.15 pg/mL	ELISA
CMTM2 (Chen et al., 2020)	Serum	China	105	86.79	88.46	0.88	–	ELISA
CSTB (Lee et al., 2008)	Serum	Korea	157	85.5	53.1	0.746	5.34 ng/mL	ELISA
CTSA (Du et al., 2020)	Serum	China	64	100	64.5	0.894	0.56 ng/mL	ELISA
CXCL13 (Li et al., 2017)	Serum	China	114	58.6	100	0.824	125.14 pg/mL	ELISA
DCD (Qiu et al., 2018)	Serum	China	164	54.88	88.64	0.769	25.75 ng/mL	ELISA
DKK1 (El-Shayeb et al., 2021)	Serum	Egypt	175	89	80	0.92	2.3 ng/mL	ELISA
MDK (El-Shayeb et al., 2021)	Serum	Egypt	175	100	90	0.95	5.1 ng/mL	ELISA
EGFL7 (Yang et al., 2021)	Serum	China	746	77.4	82.2	0.86	2,610 ng/mL	ELISA
Gal-3BP (Liu et al., 2017)	Serum	China	160	80	93.75	0.898	–	ELISA
GDF15 (Liu et al., 2015)	Serum	China	614	86.79	72.75	0.8426	1.945 ng/mL	ELISA
hCE1 (Na et al., 2013)	Plasma	Korea	84	89.2	77.7	0.918	8 ng/mL	ELISA
HSP90α (Wei et al., 2020)	Plasma	China	889	67.07	90.43	0.836	69.1 ng/mL	ELISA
Laminin-γ2 (Kiyokawa et al., 2017)	Serum	Japan	81	63	83	0.793	116.6 pg/mL	Chemiluminescent immunoassay
LTBP2 (Da Costa et al., 2015)	Plasma	France	107	100	94	0.98	27 ng/mL	ELISA
MIF (Ismail et al., 2017)	Serum	Kingdom of Saudi Arabia, Egypt	149	22.7	92.8	0.793	197.8 μ g/L	ELISA
MRPL9 (Xie et al., 2023)	Serum	China	147	76.9	91.3	0.867	1,581.3 pg/mL	ELISA
NF-κB (Ismail et al., 2015)	Plasma	Egypt	165	84.4	75.4	0.825	193.24 ng/mL	ELISA
NRP1 (Abdel Ghafar et al., 2021)	Serum	Egypt	99	72	87.8	0.801	1,418 pg/mL	ELISA
OPN (Zhu et al., 2020)	Serum	China	322	79.21	79.64	0.851	14.64 ng/mL	ELISA
Preneoplastic antigen (Yamashita et al., 2020)	Serum	Japan	141	63.8	66	0.68	5 ng/mL	ELISA
PPIH (Ye et al., 2024)	Serum	China	32	100	68.8	0.9	–	ELISA
proCTSD (Qi et al., 2014)	Serum	China	70	85	80	0.88	125 (Unit free)	Western blot
Proteasome (Henry et al., 2009)	Plasma	France	83	72	97	0.875	2,900 ng/mL	ELISA
PTX3 (Deng et al., 2020)	Serum	China	365	79.4	89.9	0.929	9.231 ng/mL	ELISA
S100A9 (Sun et al., 2016)	Serum	China	94	91	66	0.83	92.6 ng/mL	ELISA
SSA2 (Abdel Wahab et al., 2017)	Serum	Egypt	31	70.83	85.71	0.851	6 ng/mL	ELISA
Thioredoxin (Li et al., 2015b)	Serum	China	520	84.3	91.8	0.946	20.5 ng/mL	ELISA
TLN1 (Aboelfotoh et al., 2020)	Serum	Egypt	96	100	65	0.858	14 ng/mL	ELISA
VASN (Li et al., 2015c)	Serum	China	326	69	80.5	0.77	1.5061 ng/mL	ELISA
Vimentin (Sun et al., 2010)	Serum	China	108	40.91	87.5	0.69	245 ng/mL	ELISA
VWF (Liu et al., 2014)	Plasma	China	310	91.8	71	0.83	1,100 mU/mL	ELISA
VWF/ADAMTS13 ratio (Takaya et al., 2019)	Plasma	Japan	61	51	95	0.73	5.5	ELISA
A 21-antigen panel^a^(Middleton et al., 2014)	Serum	UK, Germany	192	45	92	–	–	ELISA
A UPS signature^b^(Qu et al., 2011)	Serum	USA	70	88.5	90.2	0.938	0.5 (Unit free)	Immunofluorescence assay
Combination of CCL20 and LCN2 (Du et al., 2022)	Serum	China	379	80.8	89.2	0.927	0.443 pg/mL	ELISA
Combination of OPN, GDF15, NSE, TRAP5 and OPG (Cheng et al., 2020)	Plasma	China	418	94.49	84.76	0.896	–	Liquid chip
Combination of HABP2, CD163, AFP and PIVKA-II (Xing et al., 2023b)	Serum	China	127	0.925	0.915	0.979	–	Targeted proteomics based on parallel reaction
Combination of EID3, CNOT3 and UBE2Z (Hao et al., 2024)	Serum	China	60	0.753	–	–	–	ELISA

**Notes.**

a The panel includes AFP, Cyclin B1, Gankyrin, p53, NY-ESO-1, RalA, CK8, GRP78, HDGF, DKK1, H-RAS-1, p16, WT1, HCC1, Sui1, l-myc2, GPC-3, Beta-Catennin2, Beta-HCG, Calreticulin and FASN.

b The signature includes trypsin-like, caspase-like, chymotrypsin-like, normalized chymotrypsin-like activities of proteasomes, AFP and DCP; Sen, sensitivity; Spe, specificity; UK, United Kingdom; USA, United States of America.

Most studies provided clear results for the sensitivity, specificity and receiver operating characteristic curve (ROC) analysis of the biomarkers (Table 1). It should be noted that the results of the ROC analysis of the biomarker may be different depending on the control group selected. For example, the sensitivity, specificity and area under curve (AUC) of the biomarker are slightly changes when the control group is patients with cirrhosis or healthy people. Many studies have provided multiple ROC analysis results based on different controls. This review only presents research results with a large sample size for researchers and clinical doctors to consult.

The screening process of biomarkers usually includes three parts: training, test, and validation. The findings presented in this review are generally obtained through the training dataset as the sample size of the training set is usually large. Hepatitis B or C viruses infections are the major risk factors for the initiation of HCC, so nearly all studies recruited HCC patients with HBV or HCV. Some studies have also provided ROC analysis results comparing with AFP or in combination with AFP for diagnosis. These biomarkers often play a better diagnostic role when used in combination with AFP (Middleton et al., 2014; Qu et al., 2011).

Prognostic biomarkers mainly focused on the survival outcomes of HCC patients after treatment, especially after surgery. The predictive role of good or poor prognosis for each increased biomarker was showed by Kaplan–Meier survival curve or multivariable Cox regression analysis (Table 2). The approval of sorafenib as first-line targeted therapy for advanced HCC signified the advent of the era of systemic therapy, which targets the RAF-MEK-ERK cascade and angiogenesis *via* vascular endothelial growth factor receptor 2 (VEGFR2) (Donne & Lujambio, 2023). Therefore, the protein biomarkers used to monitor the efficacy of sorafenib are also one of the hotspots in prognostic research.

**Table 2:** Tumor-associated proteins identified for prognosis of HCC.

Biomarkers	Source	Sample origin	*N*	Treatment	Prognosis (high level)	Cutoff	End point	Method
90K/MAC-2BP (Iacovazzi et al., 2003)	Serum	Italy	36	NA	Poor	14 ng/mL	OS	ELISA
ACTR3 (Shuen et al., 2022)	Extracellular vesicles	Singapore	45	Sorafenib and selective internal radiation	Good	–	OS	LC/MS
AREG (Godin et al., 2019)	Serum	France	55	Sorafenib	Good	5% decreases	OS	ELISA
Artemin (Han et al., 2018)	Serum	China	260	Surgery	Poor	Median value	DFS	ELISA
ASGPR (Mu et al., 2014)	Circulating tumor cells	China	32	NA	Poor	–	OS	Immunofluorescence staining
CD147 (Lee et al., 2016)	Plasma	Australia	110	Locoregional therapy or sorafenib	Poor	24 ng/mL	90-day and 180-day OS	ELISA
CD24 (Maimaitiming et al., 2020)	Plasma	India	86	Surgery	Poor	7.83 ng/mL	OS, RFS	ELISA
GSN (Hu et al., 2024)	Serum	China	126	Surgery	Good	–	Early RFS	DIA-MS
HGF (Adachi et al., 2019)	Serum	Japan	80	Sorafenib	Poor	1,449.3 pg/mL	OS	Liquid chip
HIF-1α (El Shorbagy et al., 2021)	Plasma	Egypt	80	Sorafenib or sorafenib and metformin combination therapy	Poor	186 pg/mL	OS	ELISA
VEGF (El Shorbagy et al., 2021)	Plasma	Egypt	80	Sorafenib or sorafenib and metformin combination therapy	Poor	489 pg/mL	OS	ELISA
IL-18 (Tangkijvanich et al., 2007)	Serum	Thailand	70	Surgery or TACE or untreated	Poor	10^5^ pg/mL	OS	ELISA
IL-6 (Jang et al., 2012)	Serum	Korea	110	TACE	Poor	10 pg/mL	OS	ELISA
IL-8 (Ren et al., 2003)	Serum	China	59	Surgery	Poor	17.6 pg/mL	OS, DFS	ELISA
MICA (Li et al., 2013a)	Serum	China	60	TACE	Poor	1 ng/mL	OS	ELISA
PD-1 (Chang et al., 2019)	Serum	China	120	Surgery	Good	11.2 μ g/mL	OS	Antibody array assay
PD-L1 (Chang et al., 2019)	Serum	China	120	Surgery	Poor	33.0 μ g/mL	OS, DFS	Antibody array assay
ULBP1 (Easom et al., 2020)	Serum	UK	115	Surgery or transplantation or TACE or systemic chemotherapy or untreated	Poor	2000 pg/mL	OS	ELISA
Combination of LRG1, APCS, BCHE, C7, FCN3, tumor number, PIVKA and AFP (Yu et al., 2017)	Serum	Korea	180	TACE	Poor	–	PFS, OS	ELISA

**Notes.**

OS overall survival DFS disease-free survival RFS recurrence-free survival DIA-MS data-independent acquisition mass spectrometry LC-MS liquid chromatography-tandem mass spectrometry UK United Kingdom NA not available TACE Transarterial chemoembolization

There are also some biomarkers that have both diagnostic and prognostic roles in HCC (Table 3). However, there were inconsistencies in the methods used to assess the diagnostic and prognostic roles of certain biomarkers. For example, ELISA was used to evaluate the diagnostic role of MFSD2A in serum, while immunohistochemistry is used to assess the prognostic role of MFSD2A in HCC tissue (Xing et al., 2019b). In addition, some studies utilized Barcelona Clinic Liver Cancer (BCLC) or Tumor-Node-Metastasis (TNM) clinical staging systems as the disease outcome to evaluate the prognostic role of protein biomarkers, such as clusterin (Nafee et al., 2012).

**Table 3:** Tumor-associated proteins identified for both diagnosis and prognosis in HCC.

Biomarkers	Source	Sample origin	Sen (%)	Spe (%)	AUC	Prognosis (high level)	Treatment	Event	Method
14-3-3 beta (Lin et al., 2017)	Serum	China	91.4	75.3	0.798	Poor	Surgery	OS, DFS	ELISA
ADAMTS13 (Ikeda et al., 2011)	Plasm	Japan	–	–	–	Poor	Radiofrequency ablation	RFS	ELISA
Alpha-1-antitrypsin (Comunale et al., 2010; Pirisi et al., 1996)	Serum	USA/ Italy	70	86	0.867	Poor	Surgery or PEI or TACE or supportive therapy	OS	Lectin-FLISA/radio-immunoassay
ANG-2 (Abdel Ghafar et al., 2021 and Adachi et al., 2019)	Serum	Egypt/ Japan	60	73.5	0.748	Poor	Sorafenib	OS	ELISA/liquid chip
ANGPTL6 (Hu et al., 2021)	Serum/ HCC tissues	China	92.6	69.6	0.826	Poor	Surgery	OS	ELISA/LC–MS/MS
ANXA3 (Ma et al., 2018)	Serum	China	59.24	90.33	0.869	Poor	TACE and surgery therapy	OS	ELISA
Cathepsin D (Chuaypen et al., 2022)	Serum	Thailand	81.3	67.4	0.78	Poor	NA	OS	ELISA
CD14 (Dou et al., 2016)	Serum	China	–	–	0.868	Poor	NA	OS	ELISA
Clusterin (Nafee et al., 2012)	Serum	Egypt	90	87	0.95	Poor	NA	Progression of BCLC and TNM	ELISA
DLK1 (Li et al., 2015a)	Serum	China	34.6	93	0.604	Poor	Surgery	OS	ELISA
Irisin (Zhang et al., 2019)	Serum	China	76.9	76.5	0.8364	Good	Surgery	CCI score	ELISA
ITIH4 (Noh et al., 2014)	Serum	Korea	76	54.5	0.71	Good	NA	OS	Western blot
KLKB1 (Che et al., 2021)	Serum	China	–	–	0.766	Good	NA	OS, PFS	ELISA
MFGE8 (Shimagaki et al., 2019)	Serum	Japan	69.7	84.3	0.842	Good	Surgery	OS, DFS	ELISA
MFSD2A (Xing et al., 2019b)	Serum/HCC tissues	China	76.3	66.7	0.718	Poor	Surgery	5-year OS	ELISA/immuno histochemistry
NGAL (Dertli et al., 2020)	Serum	Turkey	88.2	77.8	0.873	Poor	Untreated	OS	ELISA
PRDX1 (Sun et al., 2014)	Serum/ HCC tissues	China	–	–	0.817	Poor	Surgery	OS, DFS	ELISA/ immunohistochemistry
PRDX3 (Shi et al., 2014)	Serum	China	85.9	75.3	0.865	Poor	Surgery	OS	ELISA
SCCA-IgM (Pozzan et al., 2014)	Serum	Italy	89	50	0.66	Poor	TACE or surgery or percutaneous ablation or supportive cares	OS, PFS	ELISA
Talin-1 (Mashaly et al., 2018)	Serum	Egypt	72.73	80.65	0.81	Good	NA	Portal vein invasion, metastasis and progression of BCLC	ELISA
TGFβ1 (Song et al., 2002 and Hussein et al., 2012)	Plasma/serum	Korea/ Egypt	68.4	96.8	0.9	Poor	NA	Metastasis and recurrence	ELISA

**Notes.**

Sen sensitivity Spe specificity FLISA fluorophore-linked immunosorbent assay BCLC Barcelona Clinic Liver Cancer CCI comprehensive complication index OS overall survival DFS disease-free survival RFS recurrence-free survival PFS progression-free survival USA United States of America TACE Transarterial chemoembolization PEI Percutaneous ethanol injection NA not available

## Tumor-associated Autoantibodies

TAAs can induce host immune response, leading to the generation of TAAs’ autoantibodies. These autoantibodies in the blood can be used to develop assay for detection of abnormal or dysregulated cellular processes in the tumorigenesis. TAAbs are easy to be measured as they are secreted into blood circulation, and their titers increase with the biological amplification of TAAs and their lasting immune stimulation. Additionally, unlike polypeptides, tumor-associated autoantibodies (TAAbs) are highly stable in serum and not degraded by proteases (Hong & Huang, 2015). Therefore, it is easier to detect TAAbs than TAAs themselves. The majority of the TAAbs presented in this review play a role in the diagnosis of HCC (Li et al., 2008; Heo et al., 2020; Li et al., 2024; Heo et al., 2019; Heo et al., 2010; Idriss et al., 2019; Hwang et al., 2018; Liu et al., 2012; Shen et al., 2024; Zhang et al., 2020), and only one panel of autoantibodies has the functions of and prognosis prediction of HCC simultaneously (Table 4) (Okada et al., 2020).

The screening of TAAbs can be achieved by protein microarrays technology. Protein microarrays, also known as protein chips, are solid-state surfaces (usually glass) on which thousands of proteins (such as antigens, antibodies, enzymes, substrates, *etc.*) are immobilized at different spatial locations, forming a high-density protein matrix. Protein microarrays are usually divided into two types: analytical and functional protein microarrays. Analytical protein microarrays are used to detect the expression levels of specific proteins in samples for diagnosing diseases or monitoring treatment effects. Functional protein microarrays are used to study the function and interactions of key proteins in cellular signaling pathways, in order to discover new drug targets (Hall, Ptacek & Snyder, 2007; Hu et al., 2011). So, protein microarrays provide a high throughput new method for the discovery of previously unknown multifunctional proteins and new functions of known proteins.

## Bioinformatics Analysis of Blood Protein Biomarkers in HCC

**Figure 1:**
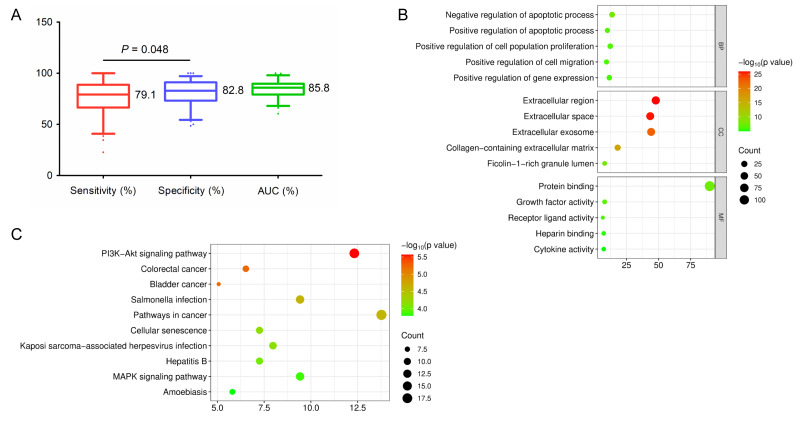
Bioinformatics analysis of blood protein biomarkers in hepatocellular carcinoma. (A) Comparison of the sensitivity, specificity and AUC of blood protein biomarkers in hepatocellular carcinoma. (B) GO analysis of blood protein biomarkers in hepatocellular carcinoma. (C) KEGG pathway analysis of blood protein biomarkers in hepatocellular carcinoma. Plots were created using GraphPad Prism software and www.bioinformatics.com.cn.

All sensitivity, specificity, and AUC values included in the study were compared, as long as the literature provided the above study results. The median sensitivity, specificity, and AUC of all blood protein markers retrieved for HCC were 79.1%, 82.8%, and 0.858, respectively. The specificity seemed to be higher than the sensitivity (*P* = 0.048) (Fig. 1A). Subsequently, these biomarkers were entered to the DAVID database (https://davidbioinformatics.nih.gov) for GO and KEGG analysis. The main biological processes (BP), molecular functions (MF), cellular components (CC) and KEGG pathways were shown in Figs. 1B and 1C. These blood biomarkers were mainly enriched in PI3K-Akt signaling pathway, pathways in cancer, cellular senescence, hepatitis B, MAPK signaling pathway, *etc*. These findings are highly corresponding to the previously reported molecular mechanisms of HCC development (Garcia-Lezana, Lopez-Canovas & Villanueva, 2021). In particular, multiple biomarkers are involved in the formation of cancer hallmarks, such as NF-κB and CCL20 are shown to be related to the tumor’s inflammatory response, HSP90α is associated with the tumor’s fatty acid metabolism, and CKAP4 is associated with IL2-STAT5 signaling pathway by retrieving the MSigDB database (https://www.gsea-msigdb.org/gsea/msigdb/index.jsp).

**Table 4:** Tumor-associated autoantibodies for diagnosis and prognosis of HCC.

Biomarkers	Sample origin	Diagnosis/ prognosis	Sample source	*N*	Sen (%)	Spe (%)	AUC	Prognosis (high level)	Method
AIF (Li et al., 2008)	China	✓/-	Serum	290	55.9	81.4	–	–	Antigen microarray
DEAD box (Li et al., 2008)	China	✓/-	Serum	290	85.6	69.8	–	–	Antigen microarray
EEF2 (Li et al., 2008)	China	✓/-	Serum	290	78.8	78.5	–	–	Antigen microarray
HNRNPA2 (Li et al., 2008)	China	✓/-	Serum	290	64.4	70.9	–	–	Antigen microarray
Prostatic binding protein (Li et al., 2008)	China	✓/-	Serum	290	48.3	82.6	–	–	Antigen microarray
TIM (Li et al., 2008)	China	✓/-	Serum	290	64.4	75	–	–	Antigen microarray
ATIC (Heo et al., 2020)	Korea	✓/-	Serum	229	70.83	90.63	0.8755	–	ELISA
BIRC5 (Li et al., 2024)	China	✓/-	Serum	86	38.5	89.4	0.716	–	Protein microarray
EIF3A (Heo et al., 2019)	Korea	✓/-	Serum	187	79.41	83.53	0.871	–	ELISA
FASN (Heo et al., 2010)	Korea	✓/-	Serum	41	96.55	100	0.9973	–	ELISA
Ku86 (Idriss et al., 2019)	Egypt	✓/-	Serum	110	94	80	0.933	–	ELISA
SF3B1 (Hwang et al., 2018)	Korea	✓/-	Serum	187	73.53	91.76	0.87	–	ELISA
Combination of CENPF, DDX3, HSPA4, HSPA5, VIM, LMNB1 and TP53 (Liu et al., 2012)	China	✓/-	Serum	190	82.86	100	0.917	–	ELISA
Combination of ZIC2, CDC37L1 and DUSP6 (Shen et al., 2024)	China	✓/-	Serum	577	56.4	82.1	0.764	–	ELISA
Combination of CIAPIN1, EGFR, MAS1, SLC44A3, ASAH1, UBL7 and ZNF428 (Zhang et al., 2020)	China	✓/-	Serum	576	68.6	92.1	0.894	–	Protein microarray
Combination of Sui1, p62, RalA, p53, NY-ESO-1 and c-myc (Okada et al., 2020)	Japan	✓/✓	Serum	234	56	91	–	Poor	ELISA

**Notes.**

Sen sensitivity Spe specificity

Diagnostic accuracy is the core of evaluating biomarkers. The accuracy of a biomarker includes sensitivity and specificity. Sensitivity represents the accuracy of the biomarker test in identifying subjects who truly have the outcome. Specificity represents the accuracy of the biomarker test in identifying subjects truly do not have the outcome. For a diagnostic test, the ideal sensitivity and specificity are both 100%, and the distribution of the test values of normal and diseased individuals not overlap at all. However, this ideal situation rarely occurs in practice, and there is usually some overlap between the test values of normal and diseased individuals. So sensitivity and specificity are inversely related, that is, as one increases the other decreases (Monaghan et al., 2021). ROC analysis provides an objective statistical mathematic method to assess the diagnostic accuracy of a test with a continuous outcome by graphically displaying the trade-offs of the true positive rate (sensitivity) and false positive rate (1-specificity) (Søreide, 2009). A good biomarker is generally considered to have at least 90% sensitivity and specificity, as well as an AUC value above 0.75 (Sarhadi & Armengol, 2022). Currently identified blood protein biomarkers could be combined into a panel to improve the sensitivity and specificity of HCC diagnosis and prognosis prediction. Of course, this requires multi-center samples and repeated trials to achieve a high AUC for clinical translation.

At present, due to the different methods of identifying potential biomarkers, it is difficult to directly compare different studies. Some biomarkers with insufficient supporting may play a key role in the diagnosis and prognostic prediction of HCC, which deserves more attention in future research.

## Discussion and Perspectives

The liver, as an organ of protein synthesis, is responsible for 85–90% of circulating proteins volume, and also secretes acute phase proteins, growth factors and many other peptides involved in regulation (Trefts, Gannon & Wasserman, 2017). These proteins also play an indicative role in the diagnosis and prognosis prediction of HCC, such as CRP (Jang et al., 2012; She et al., 2015), beta 2-microglobulin (Ouda et al., 2015), and prealbumin (Jing et al., 2020). Since these proteins are clinically common markers for assessing liver function and inflammatory response, they lack specificity for diagnosing HCC and cannot be used as independent diagnostic indicators. Although they may be associated with greater tumor burden, later staging, poorer tumor differentiation degree and shorter survival, the detection methods are unable to be unified. Therefore, these markers were not included in this review. In addition, those studies that did not provide exact ROC analysis results were also not included.

As carriers of intercellular communication, exosomes are stable and rich in composition, capable of effectively transmitting tumor information. The cargoes carried by exosomes (including proteins, RNA, DNA, lipids, *etc.*) can be released by tumor cells or tumor-associated cells. Exosomes play a critical role in the occurrence, development, metastasis, drug resistance, and immune escape of HCC by transmitting carcinogenic molecules, activating key pathways, promoting angiogenesis, driving immune evasion, and reshaping the tumor microenvironment (Zhang et al., 2024). Since exosomes have a protective membrane structure, the ncRNA carried by exosomes can escape degradation by enzymes in body fluids and maintain good stability. Therefore, the exosomal ncRNAs are very suitable as biomarkers for the diagnosis and prognosis prediction of HCC, such as exosomal miR-21-5, miR-221-3p and lncRNA-ATB (Shi et al., 2024). Due to the lack of unified exosome isolation techniques and the heterogeneity of exosomes, tumor-derived exosomes are extremely low in the blood, the proteins currently identified are mostly from HCC cell line-derived exosomes (He et al., 2015; Wang et al., 2018b; Li et al., 2019), and only a few proteins are from the blood of HCC patients (Fu et al., 2018; Arbelaiz et al., 2017; Shuen et al., 2022). Therefore, a single protein may lack sensitivity or specificity, and combining multiple membrane proteins or combining nucleic acid markers (such as miRNA) may be the key to improving diagnostic efficacy. Exosome-based diagnostics and therapeutics need to be strictly validated in clinical trials before they can be truly translated.

Circulating tumor cells (CTCs) may better reflect the characteristics of tumors that undergoing metastasis than small primary tumors. To acquire mobility and invasive ability, CTCs usually highly express stromal markers and lowly express epithelial markers. CTCs must express high levels of anti-apoptotic protein, DNA repair proteins, and activate specific survival signaling pathways to survive blood flow shear stress, immune cell attack, and anoikis. Additionally, CTCs often possess tumor stem cell characteristics, including the ability for self-renewal and unlimited proliferative capacity, which are key for colonization of new organs (Massagué & Obenauf, 2016). So the biomarker profiles in CTCs may be quite different than what exists in early tumors. However, the limited number of CTCs has restricted application to explore their genomic, transcriptomic, and proteomic characteristics. Despite this, the recent emergence of single-cell sequencing technologies has facilitated the study of the genomic and transcriptomic profiles of CTCs, while the proteomic study of CTCs remains elusive. The study of the proteome can not only provide a landscape of the biological characteristics of CTCs but also identify specific membrane proteins in CTCs (Lin et al., 2021). Therefore, the proteomic study of CTCs is urgently needed. At present, the clinical application of CTCs mainly relies on the analysis of cell count and molecular phenotype, and only a few studies have provided the changes in the expression level of protein biomarkers on the diagnosis or prognosis of HCC (Mu et al., 2014). The scarcity and difficulty in isolation of CTCs hinder their use as effective biomarkers for early cancer diagnosis, but they can be used for therapeutic monitoring and prognosis prediction (Shaik et al., 2023).

Although extensive proteomics research that has been conducted in the blood, and many potential protein biomarkers for HCC have been identified, relatively few have been ultimately applied to clinical practice. The development of HCC biomarkers from body fluids faces multiple challenges at the biological, technical and clinical levels.

The translation of the discovered biomarkers into clinical application needs to overcome several major biological barriers. First, HCC is highly heterogeneous in molecular, pathological and genetic aspects. Due to the large differences in biomarkers among different subtypes of HCC, it is difficult for a single biomarker to cover all patients (Chan et al., 2024). Second, HCC caused by different etiologies, such as viral hepatitis, alcoholic liver disease, metabolic dysfunction-associated fatty liver disease (MAFLD), may have different biomarkers. Third, the amount of biomarkers released into the blood in the early stage is extremely low and is easily masked by normal signals. Finally, chronic liver diseases, such as cirrhosis and hepatitis, may result in similar biomarker expressions, resulting in false positives.

The technical difficulties in developing liquid biopsy biomarkers for HCC include the high sensitivity and specificity requirements of detection technology. The difficulty of separating and enriching biomarkers, the extremely low number of circulating tumor cells (CTCs) in HCC, the low efficiency of exosome extraction, the short length of free DNA and the interference from background proteins. The difficulty of integrating multi-omics data, and the analytical models and standardized processes are still immature (Lu et al., 2023; Akabane et al., 2025).

Clinical validation and translation of biomarkers for HCC face difficulties. It is difficult to collect a large-scale of early-stage HCC patients, and most studies focus on advanced patients, leading to an overestimation of the efficacy of biomarkers for early diagnosis. Sample collection (*e.g.*, blood storage, temperature), detection methods, and data analysis standards are not unified, affecting the comparability of results (Ray et al., 2010). Clinical utility needs to be validated by large-scale prospective cohorts and it must be proven that new biomarkers can improve clinical outcomes independently from existing methods (Hayes, 2021). Fluid biopsy involves ethical review, and new biomarkers must pass strict pharmaceutical approval before they can be marketed.

Chronic hepatitis, liver cirrhosis, and HCC typically develop progressively. Therefore, prognostic markers for hepatitis and cirrhosis may also hold significant reference value for the diagnosis of HCC. In the future, if markers of these three diseases can be integrated into a unified framework for systematic description and comparison, it may have greater scientific significance and clinical value.

In the post-genomic era, proteomics is key to understanding systematic biology, *i.e.,* how the organisms work (Bensalah, Montorsi & Shariat, 2007). Proteome reflects the state of the organism and can thus monitor biological variations over time. These protein biomarkers could help with early diagnosis of HCC or establish tumor-specific spectra that predicts HCC prognosis. In the future, machine learning and artificial intelligence are expected to become powerful tools for integrating clinical and multi-omics data and screening panels of HCC biomarkers. These integrated molecular signatures may be involved in different mechanisms underlying the occurrence and development of HCC, thereby providing the possibility of achieving minimally invasive and dynamic monitoring, which will help in the real-time assessment of diagnosis and prognosis of HCC.

## Additional Information and Declarations

### Data Availability

The following information was supplied regarding data availability: This is a literature review.
